# Hong Kong–Macau Severe Hives and Angioedema Referral Pathway

**DOI:** 10.3389/falgy.2023.1290021

**Published:** 2023-12-06

**Authors:** Philip H. Li, Elaine Y. L. Au, Si-Leong Cheong, Ling Chung, Ka I. Fan, Marco H. K. Ho, Agnes S. Y. Leung, Martin M. H. Chung, Jane C. Y. Wong, Ricardo Coelho

**Affiliations:** ^1^ Division of Rheumatology and Clinical Immunology, Department of Medicine, The University of Hong Kong, Hong Kong, Hong Kong SAR, China; ^2^ Division of Clinical Immunology, Department of Pathology, Queen Mary Hospital, Hong Kong, Hong Kong SAR, China; ^3^ Department of Dermatology, Centro Hospitalar Conde de São Januário, Macau, Macao SAR, China; ^4^ Lee Tak Hong Allergy Centre, Hong Kong Sanatorium & Hospital, Hong Kong, Hong Kong SAR, China; ^5^ Department of Paediatrics, Faculty of Medicine, The Chinese University of Hong Kong, Hong Kong, Hong Kong SAR, China; ^6^ Hong Kong Hub of Paediatric Excellence, The Chinese University of Hong Kong, Hong Kong, Hong Kong SAR, China; ^7^ Division of Dermatology, Department of Medicine, Queen Mary Hospital, Hong Kong, Hong Kong SAR, China

**Keywords:** angioedema, hereditary, hives, Hong Kong, Macau, referral, pathway, urticaria

## Abstract

**Background:**

Urticaria (defined as the presence of hives, angioedema, or both) can be caused by a variety of etiologies ranging from more common conditions such as chronic spontaneous urticaria (CSU) to rarer conditions such as hereditary angioedema (HAE). Specialist referral may be necessary in cases of severe urticaria or HAE, but access to specialist services remains limited in certain regions, such as the Greater Bay Area (GBA) of China. To address this, the Hong Kong–Macau Severe Hives and Angioedema Referral Pathway (SHARP) was initiated by the Hong Kong Institute of Allergy and Macau Society of Dermatology to promote multidisciplinary collaboration and regional exchange of expertise in the diagnosis and management of severe urticaria.

**Methods:**

A nominated task force of dermatologists and immunologists who manage patients with severe urticaria formulated the consensus statements (CS) using the Delphi method. The consensus was defined *a priori* as an agreement of ≥80%.

**Results:**

A total of 24 CS were formulated, including four statements on classifications and definitions, seven statements on diagnosis, and 13 statements on management and referral. The definitions for acute/chronic urticaria and severe CSU were stated. Unnecessary investigations and inappropriate medications were discouraged. The characteristics and recommended approach to suspected bradykinergic angioedema were specified. Stepwise treatment options using second-generation antihistamines, omalizumab, or cyclosporin for patients with CSU were addressed, and the importance of access to HAE-specific medications was emphasized. Furthermore, an integrated referral pathway for patients with severe hives and angioedema was constructed.

**Conclusion:**

The SHARP provides guidance for the management and specialist referral of patients with severe hives and angioedema in Hong Kong and Macau.

## Introduction

Urticaria (defined as the presence of hives, angioedema, or both) remains one of the most common dermatological conditions presenting to frontline physicians and specialists alike, with a lifetime prevalence of up to 25% and 7% for hives or angioedema, respectively (1–3). Hives (also known as wheals) are characterized by pruritic and erythematous swelling of the dermis lasting typically no more than 24 h, whereas angioedema is characterized by deeper swelling involving mucosa membranes or subcutaneous tissues. Hives and/or angioedema can become chronic and caused by a wide range of differential diagnoses ranging from common and benign conditions such as chronic spontaneous urticaria (CSU) to rarer and life-threatening causes such as hereditary angioedema (HAE) (3–5). Although the majority of these conditions are caused by mast cell degranulation, isolated angioedema (i.e., angioedema without hives) may also be mediated by the accumulation of bradykinin, encompassing a group of conditions known as bradykinin-mediated angioedema (5, 6). The prototype of bradykinin-mediated angioedema is HAE, an autosomal-dominant disorder characterized by recurrent angioedema due to decreased levels of functional C1 esterase inhibitor (C1-INH), which requires disease-specific medications (7, 8). Erroneously missed or misdiagnosis of C1-INH deficiency may lead to delays in treating life-threatening angioedema or, conversely, inappropriate use and wastage of expensive medications and resources (9–13).

Regardless of etiology, the impact of uncontrolled urticaria on quality of life is often underappreciated and is comparable to that of patients suffering from rheumatoid arthritis or insulin-treated diabetes (14). Conventionally, both generalists and specialists encounter complaints of urticaria, and the majority of patients can be managed at a primary care level. However, referral to specialist care may sometimes be necessary, such as in cases of severe or difficult-to-treat CSU, suspected allergies, or bradykinin-mediated angioedema. Urticaria mimics, such as urticarial vasculitis or urticarial dermatoses (typically with lesions persisting for more than 24 h), may also benefit from specialist evaluation. Severe cases of CSU are often referred to dermatologists or immunologists, whereas cases of suspected immediate-type allergies or bradykinin-mediated angioedema are referred to immunologists. Specialists may offer immunosuppressive therapies or biologics for CSU, and specialized allergological or immunological tests are sometimes needed to exclude immediate-type allergies or confirm diagnoses of C1-INH deficiency, respectively (15). Although specialist-level care can significantly improve the management of severe CSU or C1-INH deficiency, these services and tests remain limited given the unmet provision of immunology and allergy (I&A) services in many parts of the world, including Hong Kong and Macau (10, 16, 17). Furthermore, there is a lack of clear guidance for indications or pathways for specialist management. To mitigate these shortcomings and foster multidisciplinary collaboration, various “hub-and-spoke” models have been successfully implemented in Hong Kong but have not yet been incorporated for urticaria (18–21).

Promoting cooperation and integration in healthcare services between the Greater Bay Area (GBA) of China has been advocated as an important step toward improving healthcare outcomes in the region (22). At the time of writing, Hong Kong remains the only city in the GBA with accredited specialists and training centers in both I&A and immunopathology. Despite the University of Hong Kong being recognized as an Urticaria Center of Reference and Excellence (UCARE) and Angioedema Center of Reference and Excellence (ACARE), CSU patients in Hong Kong are still likely undertreated, and the prevalence of HAE is still much lower than expected (23, 24). The situation is likely further exacerbated in Macau, with no locally diagnosed cases of HAE and a lack of accredited immunologists/allergists. To enhance inter-regional clinical services and research, the Hong Kong–Macau Severe Hives and Angioedema Referral Pathway (SHARP) was initiated by the Hong Kong Institute of Allergy (HKIA) and Macau Society of Dermatology (MSD) to aid and promote the exchange of expertise in the diagnosis and management of severe urticaria.

The primary aim of the SHARP is to help define and identify patients with severe CSU or possible bradykinin-mediated angioedema and recommend clinical pathways to optimize diagnosis and treatment for patients in Hong Kong and Macau. We present the consensus statements (CS) and clinical pathways generated by the SHARP Task Force, which provides guidance for specialist referral and principles of management for patients with severe hives and angioedema.

## Methods

CS were formulated by the SHARP Task Force using the Delphi method, which has been utilized to develop several allergy-related consensus recommendations in Hong Kong (19, 25–27). All members of the task force were nominated by the HKIA and MSD—encompassing four immunology (PL, EA, AL, and MH) and four dermatology (LC, SC, KF, and RC) representatives from Hong Kong and Macau. PL also acted as the facilitator. No ethics approval was required as no human or animal subjects were involved.

In the first Delphi round, the task force held a conference to discuss the items that should be encompassed by the SHARP. The items were discussed in three main categories relating to the clinical journey in diagnosing and managing chronic urticaria, namely, classifications and definitions, diagnosis, and management and referral. The preliminary statements were then first constructed with a range of different options available for each aspect of the CS. All members were also invited to suggest additional options if deemed necessary or more appropriate. During the second round of Delphi, all group members completed an anonymized online questionnaire to rate their agreement with each CS on a five-point Likert scale. Responses were graded as “strongly agree,” “tend to agree,” “neither agree nor disagree,” “tend to disagree,” and “strongly disagree” for each respective statement scoring +1, +0.5, 0, −0.5, and −1, respectively. The members were not required to answer all questions and could select “no opinion.” The scores were reported as a mean and standard deviation (SD). More extreme scores and lower SD indicated stronger consensus. Recommendations for each CS were subsequently drafted using standardized wording (Table 1). The consensus was defined *a priori* as agreement by at least 80% of the respondents. In the third and final rounds of Delphi, the group reviewed the aggregated questionnaire responses. If further clarification or elaboration on any statements was required, the questionnaire was adapted and returned to members with feedback.

**Table 1:** Strength of agreement and standardized wordings for levels of recommendation.

Level of recommendation	Strength of agreement	Wording
Strong recommendation	≥0.75	“We recommend”
Weak recommendation	0.50–0.74	“We suggest”
No recommendation	<0.50	“We cannot make a recommendation”

## Results

**Figure 1:**
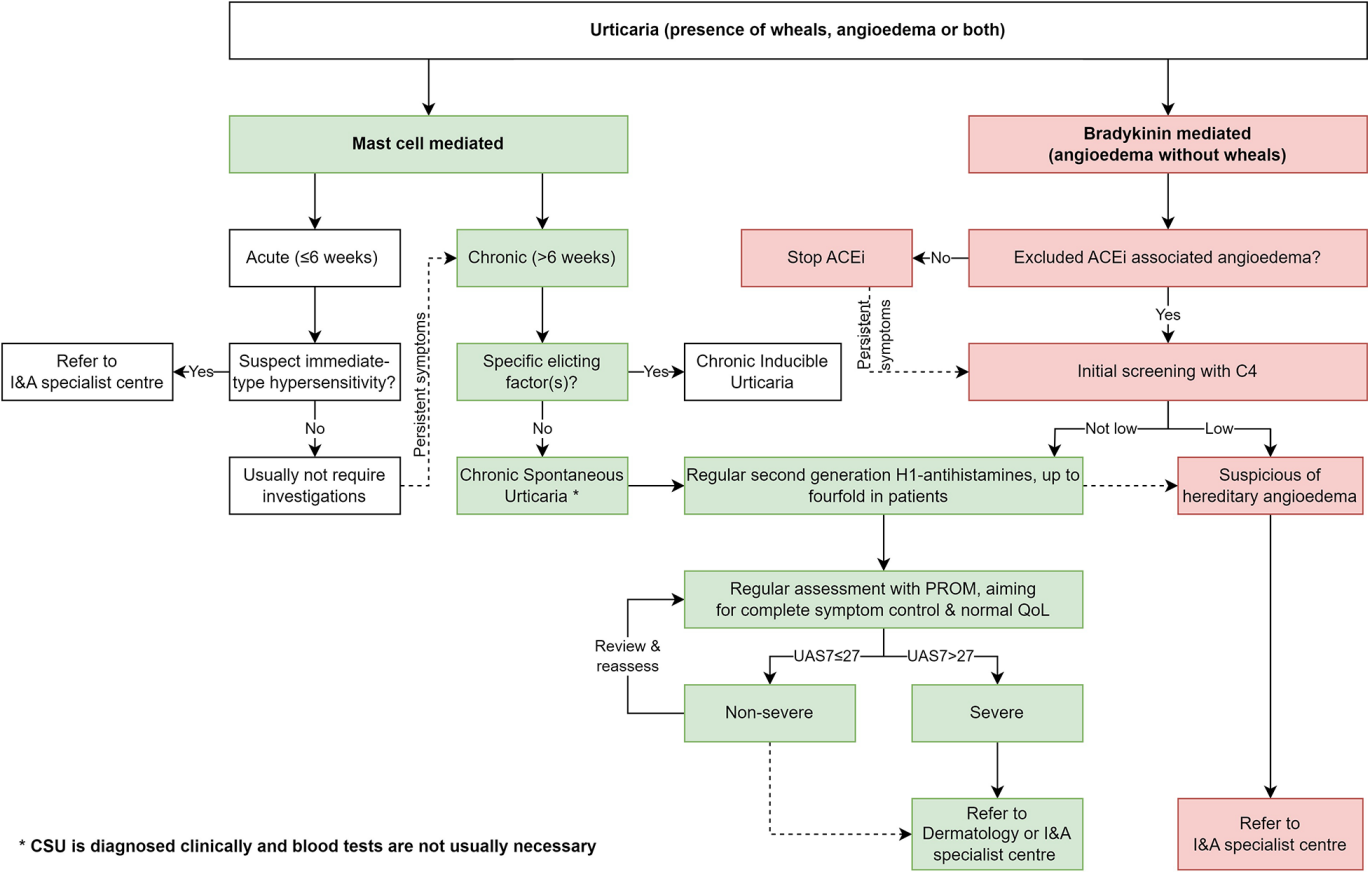
Clinical pathway for patients with severe hives and angioedema in Hong Kong and Macau.

A total of 24 CS reached consensus after multiple rounds of Delphi. A summary of the finalized CS is presented in Table 2. The clinical pathway formulated by the CS is shown in Figure 1. Detailed results of individual response weighting scores are as follows:

Classifications and definitions
  1. We suggest urticaria be characterized by wheals (hives), angioedema, or both.
    Respondent rate, 100%; agreement, 88%; strength, 0.69 ± 0.53
  2. We recommend urticaria be classified as (i) acute (≤6 weeks) or chronic (>6 weeks) and (ii) as spontaneous (absence of specific eliciting factor) or inducible (presence of specific eliciting factor).
    Respondent rate, 100%; agreement, 100%; strength, 0.88 ± 0.23
  3. We suggest “severe CSU” be defined by symptoms assessed by patient-reported outcome measures (PROM) equivalent to a weekly urticaria activity score (UAS7) above 27.
    Respondent rate, 100%; agreement, 88%; strength, 0.69 ± 0.37
Diagnosis
  4. We recommend that acute urticaria does not require routine investigations, except in the cases of suspected immediate-type hypersensitivity reactions.
    Respondent rate, 100%; agreement, 100%; strength, 0.81 ± 0.26
  5. We recommend patients with CSU be regularly assessed with PROM such as the UAS7.
    Respondent rate, 100%; agreement, 100%; strength, 0.88 ± 0.23
  6. We recommend angioedema be classified by its etiology (mast cell- or bradykinin-mediated) whenever possible (Table 3).
    Respondent rate, 100%; agreement, 100%; strength, 0.81 ± 0.26
  7. We suggest CSU be diagnosed clinically and blood tests are not usually necessary unless other diagnoses are suspected.
    Respondent rate, 100%; agreement, 88%; strength, 0.69 ± 0.37
  8. We recommend against routine allergy tests and skin biopsies for patients diagnosed with CSU.
    Respondent rate, 100%; agreement, 100%; strength, 0.82 ± 0.26
  9. We recommend angiotensin-converting enzyme inhibitor (ACEI)-associated angioedema (ACEI-AE) be excluded first in all patients with angioedema of any etiology.
    Respondent rate, 100%; agreement, 100%; strength, 0.88 ± 0.23
  10. We recommend C1-INH deficiency be considered in cases of suspected bradykinergic angioedema after ACEI-AE has been excluded.
    Respondent rate, 100%; agreement, 100%; strength, 0.81 ± 0.26
  11. We recommend initial screening for low C4 levels in patients with suspected bradykinin-mediated angioedema.
    Respondent rate, 100%; agreement, 100%; strength, 0.81 ± 0.26
Management and referral
  12. We recommend the treatment aim of urticaria should be complete symptom control and normalization of quality of life.
    Respondent rate, 100%; agreement, 88%; strength, 0.81 ± 0.37
  13. We recommend second-generation H1 antihistamines be taken regularly for the treatment of CSU.
    Respondent rate, 100%; agreement, 88%; strength, 0.81 ± 0.37
  14. We recommend second-generation H1 antihistamines up to fourfold in patients with CSU unresponsive to standard doses, before consideration of other treatments.
    Respondent rate, 100%; agreement, 88%; strength, 0.81 ± 0.37
  15. We suggest against different combinations of, especially first-generation, H1 antihistamines, to be used at the same time for the treatment of urticaria.
    Respondent rate, 100%; agreement, 88%; strength, 0.56 ± 0.32
  16. We recommend against long-term use of steroids in the treatment of urticaria.
    Respondent rate, 100%; agreement, 100%; strength, 0.81 ± 0.26
  17. We recommend against the use of ACEI in patients with a history of spontaneous angioedema of any etiology.
    Respondent rate, 100%; agreement, 88%; strength, 0.75 ± 0.38
  18. We suggest against the use of antihistamines, steroids, or adrenaline in patients with confirmed bradykinergic angioedema.
    Respondent rate, 100%; agreement, 100%; strength, 0.62 ± 0.23
  19. We recommend referral to a dermatology or I&A specialist center for patients with severe CSU not responding to a fourfold dosing of second-generation H1 antihistamines.
    Respondent rate, 100%; agreement, 100%; strength, 0.94 ± 0.18
  20. We suggest omalizumab for the treatment of severe CSU unresponsive to a fourfold dosing of second-generation H1 antihistamines.
    Respondent rate, 100%; agreement, 88%; strength, 0.69 ± 0.37
  21. We suggest cyclosporin for the treatment of severe CSU unresponsive to a fourfold dosing of second-generation H1 antihistamine and omalizumab or when omalizumab is unavailable/contraindicated.
    Respondent rate, 100%; agreement, 88%; strength, 0.56 ± 0.50
  22. We recommend referral to an I&A specialist center for patients with suspected bradykinin-mediated angioedema, where ACEI-AE has been excluded.
    Respondent rate, 88%; agreement, 100%; strength, 0.86 ± 0.39
  23. We recommend all patients with confirmed HAE should have access to HAE-specific medications (Table 4).
    Respondent rate, 100%; agreement, 100%; strength, 0.88 ± 0.23
  24. We recommend against the use of non-HAE-specific medications (such as attenuated androgens, anti-fibrinolytics, and fresh frozen plasma) for the treatment and prophylaxis of HAE.
    Respondent rate, 100%; agreement, 100%; strength, 0.75 ± 0.27

**Table 2:** Summary of consensus recommendations for patients with severe hives and angioedema in Hong Kong and Macau.

Classifications and definitions
1. We suggest urticaria be characterized by wheals (hives), angioedema, or both
2. We recommend urticaria be classified as (i) acute (≤6 weeks) or chronic (>6 weeks) and (ii) as spontaneous (absence of specific eliciting factor) or inducible (presence of specific eliciting factor)
3. We suggest “severe chronic spontaneous urticaria (CSU)” be defined by symptoms assessed by patient-reported outcome measures (PROM) equivalent to a weekly urticaria activity score (UAS7) above 27
Diagnosis
4. We recommend that acute urticaria does not require routine investigations, except in the cases of suspected immediate-type hypersensitivity reactions
5. We recommend patients with CSU be regularly assessed with PROM such as the UAS7
6. We recommend angioedema be classified by its etiology (mast cell- or bradykinin-mediated) whenever possible
7. We suggest CSU be diagnosed clinically and blood tests are not usually necessary unless other diagnoses are suspected
8. We recommend against routine allergy tests and skin biopsies for patients diagnosed with CSU
9. We recommend angiotensin-converting enzyme inhibitor (ACEI) associated angioedema (ACEI-AE) be excluded first in all patients with angioedema of any etiology
10. We recommend C1 esterase inhibitor (C1-INH) deficiency be considered in cases of suspected bradykinergic angioedema after ACEI-AE has been excluded
11. We recommend initial screening for low C4 levels in patients with suspected bradykinin-mediated angioedema
Management and referral
12. We recommend the treatment aim of urticaria be complete symptom control and normalization of quality of life
13. We recommend second-generation H1 antihistamines be taken regularly for the treatment of CSU
14. We recommend second-generation H1 antihistamines up to fourfold in patients with CSU unresponsive to standard doses, before consideration of other treatments
15. We suggest against different combinations of, especially first-generation, H1 antihistamines, to be used at the same time for the treatment of urticaria
16. We recommend against long-term use of steroids in the treatment of urticaria
17. We recommend against the use of ACEI in patients with a history of spontaneous angioedema of any etiology.
18. We suggest against the use of antihistamines, steroids, or adrenaline in patients with confirmed bradykinergic angioedema
19. We recommend referral to a dermatology or I&A specialist center for patients with severe CSU not responding to a fourfold dosing of second-generation H1 antihistamines
20. We suggest omalizumab for the treatment of severe CSU unresponsive to a fourfold dosing of second-generation H1 antihistamines
21. We suggest cyclosporin for the treatment of severe CSU unresponsive to a fourfold dosing of second-generation H1 antihistamine and omalizumab; or when omalizumab is unavailable/contraindicated.
22. We recommend referral to an I&A specialist center for patients with suspected bradykinin-mediated angioedema, where ACEI-AE has been excluded
23. We recommend all patients with confirmed hereditary angioedema (HAE) should have access to HAE-specific medications
24. We recommend against the use of non-HAE-specific medications (such as attenuated androgens, anti-fibrinolytics, and fresh frozen plasma) for the treatment and prophylaxis of HAE

**Table 3:** Distinguishing features of mast cell vs. bradykinin-mediated angioedema.

	Bradykinin-mediated	Mast cell-mediated
Duration	Slow onset and resolution (usually days)	Rapid onset and resolution (usually minutes to hours)
Concomitant hives	No	Often
Gastrointestinal involvement	Yes, bowel edema leading to obstruction	Uncommon, although possible in anaphylaxis
Respiratory involvement	Yes, due to laryngeal edema	Uncommon, although possible in anaphylaxis
Family history	Yes, in hereditary angioedema	No
Response to adrenaline, H1 antihistamines, corticosteroids, or omalizumab	No	Yes
Response to icatibant or other bradykinin-targeted medications	Yes	No

**Table 4:** HAE-specific on-demand and prophylactic medications.

On-demand medications	Prophylactic medications
C1 esterase inhibitor (human)^a^	Berotralstat
C1 esterase inhibitor (recombinant)	C1 esterase inhibitor (human)^a^
Ecallantide	Lanadelumab^a,b^
Icatibant^a,b^	

^a^ Registered in Hong Kong at the time of writing.

^b^ Registered in Macau at the time of writing.

## Discussion

The CS and clinical pathway formulated for the SHARP serve as expert recommendations toward the diagnosis, management, and suggested referral pathway for patients with severe hives and angioedema in our region. Not all CS are evidence-based and are not meant to replace existing international guidelines (3, 8). Although international guidelines are an important reference to define a “universal” standard of care, many regions (especially those lacking specialist services, such as Hong Kong and Macau) cannot follow such guidelines due to limitations of resources, expertise, or infrastructure. In contrast, the SHARP reflects the collective expert opinions tailored to the locals of Hong Kong and Macau, aiming to leverage access to specialized services and research capabilities for the entire region. Furthermore, although mast cell- (such as CSU) and bradykinin-mediated angioedema (such as HAE) can both present similarly and sometimes be clinically indistinguishable, there have been very few integrated guidelines or referral pathways encompass both conditions. Most guidelines pertain to only CSU or HAE separately, but the SHARP aims to incorporate recommendations for both disease entities into one integrated clinical pathway. It is hoped the CS and clinical pathway presented by the SHARP can help frontline healthcare workers differentiate and appropriately refer cases with either severe CSU or suspected HAE.

All statements that reached consensus were also concordant with international CSU and HAE guidelines (3, 8). More recommendations were focused on the assessment and initial management of CSU in comparison to HAE, given the relatively dire consequences and necessity for immunologist input irrespective of HAE severity. The task force also agreed that non-severe CSU generally do not require specialist care, and therefore it was necessary to define “severe CSU” and recommend initial CSU treatment. For example, the use of PROM such as UAS7 in monitoring disease activity and guiding treatment was emphasized, and a UAS >27 (or equivalent) was used to define “severe” CSU, which is in line with international recommendations and the subsidy criteria for omalizumab in Hong Kong (28, 29). The task force also recommended against unnecessary investigations and inappropriate medications (such as first-generation H1 antihistamines or long-term steroids) for CSU to avoid further wastage of scarce specialist resources and expertise in the region. According to international guidelines, routine tests for CSU include differential blood count, inflammatory markers, IgG anti-TPO, and total IgE (3). However, due to the limited accessibility of certain immunological tests in our region, we recommend referral to immunologists/allergists or dermatologists for further investigations if mimickers and important differentials (including suspected immediate-type allergies) are suspected. Omalizumab, being the only licensed treatment for urticaria refractory to maximal dose H1 antihistamines, was also recommended for severe CSU. In the context of bradykinin-mediated angioedema, more emphasis was made on initial screening and diagnosis, including the exclusion of possible ACEI-AE. Thereafter, all cases of bradykinin-mediated angioedema should be referred for immunologist evaluation for possible HAE. Given the treatment of HAE in resource-poor regions has often been compromised due to the access to treatment and specialist care, the CS also highlights the importance of using specific medications for HAE patients. In the era with multiple HAE-specific on-demand and prophylactic medications available on the market, the task force recommends against the use of non-HAE-specific medications (such as attenuated androgens, anti-fibrinolytics, and fresh frozen plasma) owing to its suboptimal efficacy and undesirable side effects, unless there are no available HAE-specific on-demand medications in an emergency (30, 31). Various HAE-specific medications have been registered in both Hong Kong and Macau, although only on-demand therapies are currently subsidized (in Hong Kong only). In contrast, prophylactic treatment is still only available under compassionate use or through clinical trials (32). The expert panel strongly recommends that all patients with HAE should have access to HAE-specific medications (as detailed in Table 4) and healthcare authorities should recognize the importance of access to these life-saving medications as there are no better substitutes.

The potential for bias in the task force still cannot be ignored. The members were nominated by HKIA and MSD as a diverse group of professionals, including dermatologists, clinical immunologists and allergists, immunology pathologists, and pediatric immunologists. This deliberate selection aimed to improve the representativeness of the task force, but potential bias could not be completely excluded. Second, the evidence grading does not signify a disregard for evidence-based practices. Although the emphasis is placed on expert judgment and collective decision-making, the Delphi method aims to address gaps in knowledge and offer practical recommendations. The SHARP also represents the first step in collaboration between dermatologists and immunologists in the GBA and due to resource and expertise disparities, not all available evidence may be available or applicable in this setting. Future longitudinal studies will be required to review the utility and outcomes of the SHARP. Studies to determine whether this pathway is translatable for other clinicians who see hives and angioedema, such as general practitioners, family medicine physicians, otorhinolaryngologists, gastroenterologists, and emergency physicians.

Overall, the SHARP intends to encourage close collaboration between dermatologists and immunologists and is an important step toward integrating healthcare in the GBA to improve healthcare outcomes for the region. By collaborating and sharing resources, Hong Kong and Macau can provide more comprehensive and coordinated healthcare services to their growing population, foster regional collaboration, and become a hub for medical research and innovation. In addition, it is hoped that SHARP will serve as a model and foundation for further collaboration and initiatives among cities of the GBA.

## Data Availability

The raw data supporting the conclusions of this article will be made available by the authors, without undue reservation.
